# A High-Fat, High-Cholesterol Diet Promotes Intestinal Inflammation by Exacerbating Gut Microbiome Dysbiosis and Bile Acid Disorders in Cholecystectomy

**DOI:** 10.3390/nu15173829

**Published:** 2023-09-01

**Authors:** Fusheng Xu, Zhiming Yu, Yaru Liu, Ting Du, Leilei Yu, Fengwei Tian, Wei Chen, Qixiao Zhai

**Affiliations:** 1 State Key Laboratory of Food Science and Resources, Jiangnan University, Wuxi 214122, China; 6210112164@stu.jiangnan.edu.cn (F.X.); 6210113061@jiangnan.edu.cn (Y.L.); 1012200610@stu.jiangnan.edu.cn (T.D.); edyulei@126.com (L.Y.); fwtian@jiangnan.edu.cn (F.T.); chenwei66@jiangnan.edu.cn (W.C.); 2 School of Food Science and Technology, Jiangnan University, Wuxi 214122, China; 3 Wuxi People’s Hospital Afliated to Nanjing Medical University, Wuxi 214023, China; yuzhimingxinjie@sina.com; 4 National Engineering Research Center for Functional Food, Jiangnan University, Wuxi 214122, China

**Keywords:** post-cholecystectomy, gut microbiota, bile acids, diet, cholesterol, inflammation

## Abstract

Patients with post-cholecystectomy (PC) often experience adverse gastrointestinal conditions, such as PC syndrome, colorectal cancer (CRC), and non-alcoholic fatty liver disease (NAFLD), that accumulate over time. An epidemiological survey further revealed that the risk of cholecystectomy is associated with high-fat and high-cholesterol (HFHC) dietary intake. Mounting evidence suggests that cholecystectomy is associated with disrupted gut microbial homeostasis and dysregulated bile acids (BAs) metabolism. However, the effect of an HFHC diet on gastrointestinal complications after cholecystectomy has not been elucidated. Here, we aimed to investigate the effect of an HFHC diet after cholecystectomy on the gut microbiota–BA metabolic axis and elucidate the association between this alteration and the development of intestinal inflammation. In this study, a mice cholecystectomy model was established, and the levels of IL-Iβ, TNF-α, and IL-6 in the colon were increased in mice fed an HFHC diet for 6 weeks. Analysis of fecal BA metabolism showed that an HFHC diet after cholecystectomy altered the rhythm of the BA metabolism by upregulating liver CPY7A1, CYP8B1, and BSEP and ileal ASBT mRNA expression levels, resulting in increased fecal BA levels. In addition, feeding an HFHC diet after cholecystectomy caused a significant dysbiosis of the gut microbiota, which was characterized by the enrichment of the metabolic microbiota involved in BAs; the abundance of pro-inflammatory gut microbiota and related pro-inflammatory metabolite levels was also significantly higher. In contrast, the abundance of major short-chain fatty acid (SCFA)-producing bacteria significantly decreased. Overall, our study suggests that an HFHC diet after cholecystectomy promotes intestinal inflammation by exacerbating the gut microbiome and BA metabolism dysbiosis in cholecystectomy. Our study also provides useful insights into the maintenance of intestinal health after cholecystectomy through dietary or probiotic intervention strategies.

## 1. Introduction

The gallbladder (GB) is primarily responsible for the storage and concentration of bile acids (BAs) secreted by the liver. Generally, GB contraction is stimulated by cholecystokinins secreted through the duodenum during the interdigestive period to expel bile [1]. Bile salts and BAs facilitate the absorption of dietary lipids [2,3]. Post-cholecystectomy (PC) can alter the dynamic balance of the BA metabolism, resulting in increased BA reabsorption and enterohepatic circulation [4]. According to epidemiological surveys, PC increases the risk of colorectal cancer (CRC) [5] and non-alcoholic fatty liver disease (NAFLD) [6], but further studies are needed to confirm the relationship, as well as to clarify the mechanism of action. Studies have shown that PC can disrupt gut microbiota homeostasis and perturb the bile acid metabolism [7], a potential risk after cholecystectomy. Moreover, the risk of cholecystectomy is often associated with the excessive intake of a high-fat, high-cholesterol (HFHC) diet [8,9] that further disrupts gut microbial homeostasis and the BA metabolism after cholecystectomy [10,11].

The gut microbiota metabolism is an important microbial pathway for the BA metabolism and is involved in the conversion of unconjugated and secondary BAs. Therefore, the composition of the gut microbiota is essential for maintaining a stable BA pool, which has a positive effect on host health [12]. Specifically, the modification of primary BAs by the gut microbiota, mainly through four metabolic pathways (ncoupling, dehydroxylation, oxidation, and exosomalization) increases the diversity of the BA pool and overall hydrophobic primary BAs [13]. In contrast, BAs, as an antimicrobial agent, can affect gut microbial homeostasis directly and through indirect effects [14]. Bacteria containing BA-metabolizing enzymes such as bile salt hydrolase (BSH) are favored because they are tolerant to the toxic effects of BAs [15]. In addition to their digestive role, BAs act as important signaling molecules that can significantly influence pathways such as metabolism and immunity in the host [16]. First, the strong hydrophobicity of BAs can exert carcinogenic effects by damaging the cells and inducing inflammation [17]. Additionally, BAs can indirectly affect the intestinal inflammatory milieu by activating or inhibiting BA receptors, such as FXR and TGR5 [16].

Dietary patterns have attracted widespread attention as a key factor affecting health. Diets rich in saturated fats and cholesterol, often associated with the Western diet [18], and malnutrition resulting from imbalanced energy or nutrient intake have been linked to the development of a variety of inflammatory diseases, such as inflammatory bowel disease, ulcerative colitis, and Crohn’s disease [19]. In addition, diet is a key factor influencing gut microbial homeostasis and the BA metabolism. Primarily, fat and fiber intake can significantly alter the gut microbiota and BA metabolism [20]. Cholesterol is an important prerequisite for BA synthesis, and chronic HFHC intake can upregulate the BA synthesis pathway, thereby increasing overall host BA levels [21]. Diets rich in dietary fiber help to maintain intestinal flora diversity and normal metabolic function [22]. The gut microbiota ferments dietary fiber to produce short-chain fatty acids (SCFAs). SCFAs play an important role in maintaining intestinal barrier function; in addition, SCFAs can reduce oxygen levels in the intestinal lumen and maintain normal immunometabolic pathways [23]. In addition, an HFHC diet can affect intestinal health by increasing intestinal barrier permeability and disrupting gut microbial homeostasis [24]. Thus, diverse dietary intake can influence host health by influencing the gut microbiota–BA axis.

High-fat and high-cholesterol diets have been widely used to simulate mice models of diseases, such as obesity [25], NAFLD [26], and hypercholesterolaemia [27], caused by excessive fat and cholesterol intake. Based on these findings, we hypothesized that an HFHC diet after cholecystectomy may exacerbate the dysbiosis of gut microbial homeostasis and BA metabolic rhythms in cholecystectomy, thereby promoting intestinal inflammation. We established a cholecystectomized mice model and fed an HFHC diet for 6 weeks. Systematic investigations were conducted based on the changes in intestinal histopathology, fecal BA metabolism, BA-related gene expression, intestinal microbiota composition, and metabolic pathways to explore the effects of an HFHC diet on the BA metabolism and microbiota balance in cholecystectomy. The results of our study provide evidence that an HFHC diet after cholecystectomy promotes the development of colonic inflammation and its associated mechanisms to provide new and valuable insights into the prevention of related diseases after cholecystectomy through dietary strategies or probiotic supplementation strategies.

## 2. Materials and Methods

### 2.1. Animals and Feeding

The C57BL/6J mice PC model has been widely used in various studies [7]. Therefore, C57BL/6J mice were used in this study for experiments. C57BL/6J mice were of specific-pathogen-free (SPF) grade (6–8 weeks, male) (SPF Biotechnology, Beijing, China). Mice were housed in a 12 h diurnal cycle at a constant temperature and humidity of 22 ± 1 °C and 50 ± 10%, respectively.

In order to simulate the two dietary patterns of HFHC and low fat and low cholesterol (LFLC) after cholecystectomy, we established a higher level of cholesterol intake in the experimental group under the premise of a reasonable combination of various nutrients. HFHC group: feeding an irradiation-sterilized HFHC diet (60% of energy from fat, 26% from carbohydrate, and 14% from protein; added an extra 1.8% of cholesterol; TP 23400-180, Trophic Animal Feed High-Tech Co., Ltd., Nantong, China). LFLC group: feeding an irradiation-sterilized LFLC diet (10% energy from fat, 76% from carbohydrates, and 14% from protein; added an extra 0.2% of cholesterol; TP 23402-020, Trophic Animal Feed High-Tech Co., Ltd., Nantong, China). Refer to Table S1 for feed formulation details. This study was approved by the Experimental Animal Ethics Committee of Jiangnan University (Qualification number: JN. No20220930c0550401[403]).

In the PC group, the GB duct was ligated, and the GB was removed after bile emptying. Mice in the negative control group underwent sham surgery (NC).

**Figure 1:**
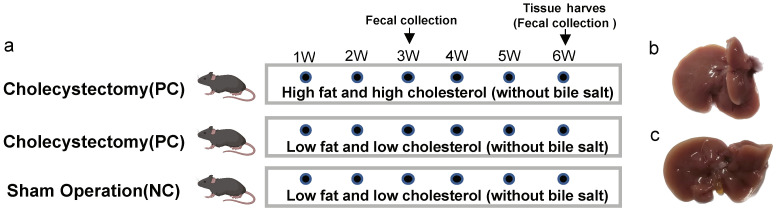
Animal experiments (**a**). Mice grouping instructions. (**b**,**c**). Diagram of cholecystectomy (**b**) with sham surgery (**c**).

After one week of acclimation, the mice were randomly divided into three treatment groups: cholecystectomy and sham surgery. As shown in Figure 1a, the experimental groups were as follows: (1) cholecystectomy and HFHC feeding (Figure 1b) (HFHC-PC) (*n* = 9); (2) cholecystectomy and LFLC feeding (Figure 1b) (LFLC-PC) (*n* = 9); and (3) sham operation and LFLC feeding (Figure 1c) (LFLC-NC) (*n* = 9).

### 2.2. Histological Analysis

Colon tissue specimens were end-fixed in 4% paraformaldehyde for more than 24 h, and sections were eventually stained with hematoxylin and eosin (H&E) and examined microscopically [28]. Please refer to the Supplementary Material for the detailed procedure.

### 2.3. Microbiome Analysis

Please refer to the Supplementary Material for the detailed sequencing procedure for fecal 16S-RNA. Briefly, Microbial Ecology Quantitative Analysis Platform 2 (QIIME2) was used to analyze the raw sequencing data [29]. First, raw sequencing data were quality filtered and demultiplexed using the DADA2 package of the QIIME2 platform. Reads were assigned using open-reference amplicon sequence variants (ASVs). Finally, the Silva Bacterial Database was used for sequence alignment. GraphPad Prism 8 and R were used for data analysis and visualization, and Python NumPy and SciPy libraries were used for ASV tracking. Microbial co-abundance analysis was performed using the Chiplot website chiplot (https://www.chiplot.online/#Bar-plot, accessed on 15 April 2023). Spearman correlation, heatmap analysis, and visualization were performed using OmicStudio tools (https://www.omicstudio.cn/tool, accessed on 7 June 2023). Kyoto Encyclopedia of Genes and Genomes (KEGG) and KEGG orthology (KO) enrichment analysis and pathway annotation were based on the Gene Denovo (https://www.genedenovo.com/, accessed on 7 June 2023) and KEGG pathway database (http://www.genome.jp/kegg/, accessed on 7 June 2023), respectively.

### 2.4. BA Measurements

Liquid chromatography–tandem mass spectrometry was used to determine the absolute abundance of the target BAs [30]. For sample pre-treatment, freeze-dried fecal samples were weighed (approximately 50 mg). Grinding and homogenization were performed using methanol (100%). A 0.22 μm membrane was used for filtration and stored in an injection bottle for LC-MS analysis.

UPLC-Q Exactive system (UPLC: UltiMate 3000); column: ACQUITY UPLC^®^ HSS T3 (1.8 µm, 2.1 × 100 mm) was used for quantitative analysis of BAs. An aqueous solution of 1 mM ammonium acetate (phase A) and a methanolic solution of 1 mM ammonium acetate (phase B) were used for elution. Gradient elution conditions are listed in Table S2.

### 2.5. RNA Extraction and Quantitative Real-Time PCR (RT-qPCR)

Total RNA was extracted using the Total RNA Isolation Kit (Vazyme, R401-01, Nanjing, China) and reverse transcribed into cDNA using HiScript III Reverse Transcriptase (Vazyme #R333, Nanjing, China). RT-qPCR was performed to detect the corresponding gene expression. The relative level of change in target genes was calculated using 2^−ΔΔCT^. Please refer to Table S3 for the primers used for RT-qPCR.

### 2.6. Analysis of Cytokines in the Colonic Tissues by Enzyme-Linked Immunosorbent Assay (ELISA)

ELISA kit (Valukine ELISA) (R&D Systems, Shanghai, China) was used for the detection of IL-1β and TNF-α levels in the colon.

Bicinchoninic Acid Assay kits (Beyotime Biotechnology, Shanghai, China) were used to determine the total protein content of all colonic tissues [31].

### 2.7. Untargeted Metabolomics

A UIUI3000 high-performance liquid chromatography (HPLC) system (Thermo Fisher Technologies, Waltham, MA, USA) was used in conjunction with a high-resolution Q Active Mass Spectrometer (Thermo Fisher Technologies, Waltham, MA, USA) to analyze fecal metabolites. The preparation methods of the metabolites, analytical parameters of HPLC-MS, and analytical methods for the metabolite data are described in detail in the Supplementary Material.

### 2.8. Determination of Plasma Biochemical Indices

The plasma was stored at −80 °C. After thawing, 80 μL of plasma was collected and diluted to 240 μL with 0.9% normal saline. Blood total cholesterol (TC), triglyceride (TG), high-density lipoprotein cholesterol (HDL-C), and glucose (Glu) levels were determined using an automatic biochemistry analyzer [32].

### 2.9. Determination of Fecal SCFA Contents

Analysis of SCFA levels in samples was performed using gas chromatography–mass spectrometry (GC-MS) [33]. The sample processing method was described by Wang et al. [34]. Detailed parameters for GC-MS testing are provided in the Supplementary Material.

### 2.10. Statistical Analysis

Statistical analysis of the data was performed using R or GraphPad Prism 8. Data are expressed as median or means ± SEM. The *t*-test, ANOVA, and Mann–Whitney test were used to test for differences in the data. Statistical significance was set at *p* < 0.05. The *p*-values in the figure represent the following: ns, *p* > 0.05 (not significant, may not be indicated); * *p* < 0.05; ** *p* < 0.01; *** *p* < 0.001; and **** *p* < 0.0001.

## 3. Results

### 3.1. Pro-Inflammatory Effects of Cholecystectomy on the Intestine Exacerbated by an HFHC Diet

**Figure 2:**
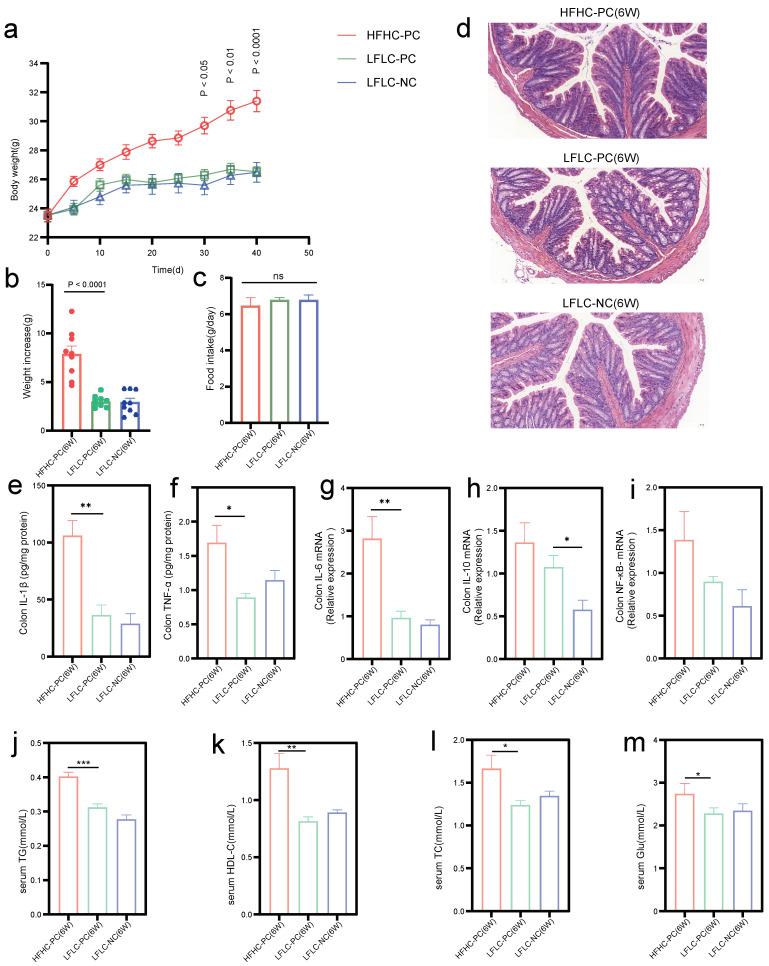
HFHC feeding and PC induce an inflammatory colonic environment in mice. (**a**): Changes in body weight of mice throughout surgical molding and different dietary feeding. (**b**): Weight increase in mice from week 1 to 6. (**c**): Mean food intake of mice throughout the experimental cycle. (**d**): Representative hematoxylin-and-eosin-stained sections in HFHC−PC, LFLC−PC, and LFLC−NC groups at week 6. (**e**,**f**): ELISA assay was used to detect IL-1β and TNF-α levels in the colon. (**g**–**i**): Relative mRNA expression of IL-6, IL-10, and NF-κB in the colon. (**j**–**m**): Serum TG, HDL-C, TC, and Glu levels. * *p* < 0.05, ** *p* < 0.01, *** *p* < 0.001, ns, nonsignificant.

We first examined whether cholecystectomy and feeding an HFHC diet after cholecystectomy resulted in changes in serum biochemistry, histology, and expression levels of inflammatory cytokines. The results showed that all three groups of mice gained weight during the experimental period (Figure 2b), with a significant difference in weight gain on day 30 in the HFHC-PC group compared to the LFLC-PC group (*p* < 0.05) (Figure 2a). However, there was no significant difference in food intake (Figure 2c). Similarly, we observed significantly increased serum TG (*p* < 0.001), HDL-C (*p* < 0.01), TC (*p* < 0.05), and Glu (*p* < 0.05) levels in the HFHC-PC group (Figure 2j–m). Compared to the LFLC-NC group, the LFLC-PC group showed a significant increase in IL-10 (*p* < 0.05) level, whereas IL-1β, IL-6, and NF-κB only showed an upward trend. In addition, compared to the LFLC-PC group, the HFHC-PC group showed increased levels in colonic cytokines IL-1β (*p* < 0.01), TNF-α (*p* < 0.05), IL-6 (*p* < 0.01), IL-10, and NF-κB (Figure 2e–i), although there was no significant tissue damage (Figure 2d). Thus, feeding an HFHC diet for 6 weeks after cholecystectomy elevated the serum levels of glycolipid metabolism in mice. It also resulted in increased expression of colonic inflammatory factors.

### 3.2. HFHC Diet Exacerbates the Dysbiosis of Gut Microbial Homeostasis in Cholecystectomy

**Figure 3:**
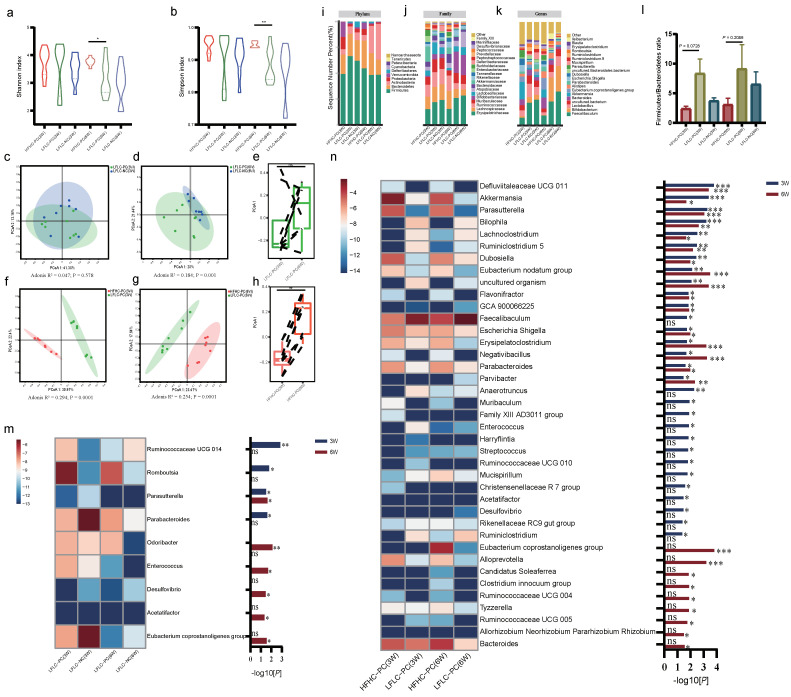
HFHC diet and PC altered gut microbial homeostasis. (**a**,**b**): Shannon or Simpson index of fecal microbiota (weeks 3 and 6). (**c**,**d**): Microbial clustering based on Bray Curtis distances in the LFLC−PC and LFLC−NC groups at weeks 3 (**c**) and 6 (**d**), visualized using principle coordinate analysis (PCoA). (Adonis; R2 = 0.047, *p* = 0.578 at week 3; R2 = 0.184, *p* = 0.001 at week 6; LFLC−PC vs. LFLC−NC). (**f**,**g**): Microbial clustering based on Bray Curtis distances in the HFHC−PC and LFLC−PC groups at weeks 3 (**f**) and 6 (**g**), visualized using principle coordinate analysis (PCoA) (Adonis; R2 = 0.294, *p* = 0.0001 at week 3; R2 = 0.254, *p* = 0.0001 at week 6; HFHC−PC vs. LFLC−PC). (**e**,**h**): Boxplot showing changes in microbiome from weeks 3 to 6, in the HFHC−PC (**h**) andLFLC−PC (**e**) groups. In both groups, the HFHC diet resulted in an increase along the first principal axis (** *p* < 0.01; ns, nonsignificant). (**i**–**k**): The relative abundance of gut microbiota at Phylum (**i**), Family (**j**), and Genus (**k**) levels from different groups (the HFHC−PC, LFLC−PC, and LFLC−NC groups). (**l**): Firmicutes/Bacteroidetes ratio. (**m**,**n**): Heat-map analysis of bacteria in fecal samples from cholecystectomized (**m**) and differentially diet-fed (**n**) mice at weeks 3 and 6, respectively. The color indicates the median relative abundance of bacteria in that group of samples. The significance of the difference in bacteria between the two groups is shown on the right. The bar color indicates the statistical significances (*p* value, values converted by −log10) of bacteria between two groups. * *p* < 0.05, ** *p* < 0.01, *** *p* < 0.001, ns, nonsignificant. The two groups were compared using Mann–Whitney test.

The gut microbial–BA axis is an important pathway for the metabolism of BAs in the colon, further modifying primary BAs to secondary BAs. The fecal microbiota was also identified. Cholecystectomy did not alter the alpha diversity of gut microbiota (Figure 3a,b). Feeding an HFHC diet for 6 weeks after cholecystectomy resulted in a significant increase in alpha diversity (Figure 3a,b). PCoA showed that the LFLFC-PC and LFLC-NC groups were significantly different at week 6 (but not at week 3) (Figure 3c,d), whereas the HFHD-PC and LFLC-PC groups were significantly different at both weeks 3 and 6 (Figure 3f,g). Mice fed an HFHC diet after cholecystectomy also transitioned along with the first principal coordinates from weeks 3 to 6, whereas mice after cholecystectomy did not follow the same gut microbiota transition (Figure 3e,h). Stacked histograms show changes in the gut microbiota at the phylum (Figure 3i), family (Figure 3j), and genus levels (Figure 3k). Notably, at weeks 3 and 6, the HFHC-PC group Firmicutes/Bacteroidetes abundance ratio showed a decreasing trend, which was negatively correlated with obesity (Figure 3l). Furthermore, we identified bacteria with significant differences at the genus level (Figure 3m,n). In the analysis of the gut microbial co-abundance network, the HFHC-PC group showed more complex relationships with increasing time than the LFLC-PC group (Figure S1a–d). Compared with the LFLC-NC group, the LFLC-PC group was significantly enriched in *Ruminococcaceae UCG 014* and *Romboutsia* at week 3, whereas *Parasutterella* and *Parabacteroides* showed a decreasing trend in abundance. At week 6, *Parasutterella*, *Odoribacter*, and *Enterococcus* were significantly enriched, whereas *Desulfovibrio*, *Acetatifactor*, and *Eubacterium coprostanoligenes groups* showed a decreasing trend (Figure 3m). Feeding an HFHC diet after cholecystectomy resulted in more complex microbiota differences. At week 3 or 6, the HFHC-PC group was highly enriched in several genera involved in BA metabolism, such as *Akkermansia*, *Parasutterella*, *Dubosiella*, *Bacteroides*, *Parabacteroides,* and *Family XIII AD3011 group*, etc. (Figure 3n). In addition, *Eubacterium nodatum group*, *Flavonifractor*, *Erysipelatoclostridium*, *Negativibacillus*, *Muribaculum*, *Clostridium innocuum group*, and *Tyzzerella* were also significantly enriched in the HFHC-PC group (Figure 3n). In contrast, *Lachnoclostridium*, *Ruminiclostridium 5*, *Fecalibaculum*, *Anaerotruncus*, *Ruminococcaceae UCG 010*, and *Ruminiclostridium* exhibited decreasing trends (Figure 3n). The results indicated that PC can affect gut microbiota homeostasis through time accumulation. In addition, feeding an HFHC diet after cholecystectomy exacerbates the disruption of gut microbiota homeostasis by cholecystectomy, resulting in alterations in the structure of gut microbiota diversity and differential bacterial profiles.

### 3.3. Disturbed BA Metabolism Attributed to Cholecystectomy Exacerbated by HFHC Diet

**Figure 4:**
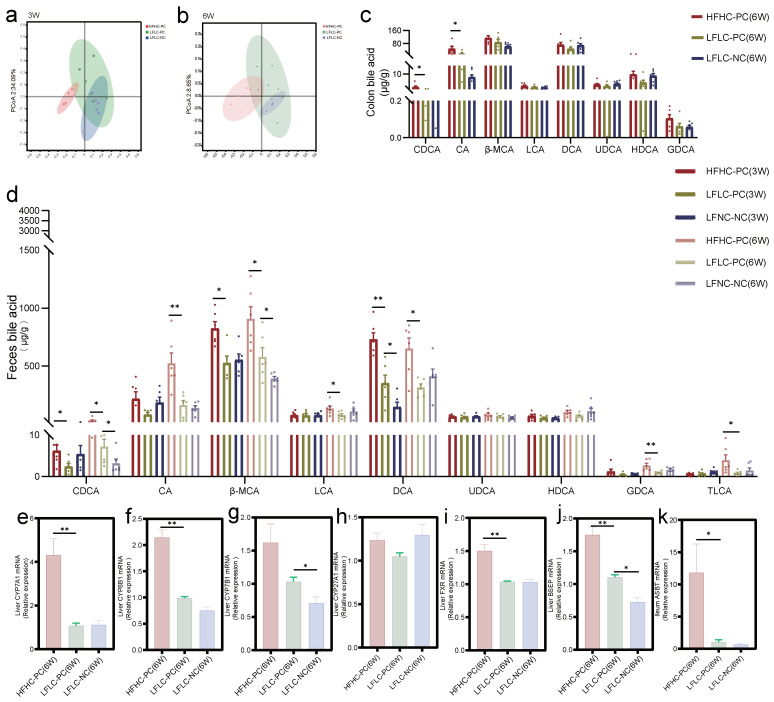
HFHC diet and PC altered BAs metabolic homeostasis. (**a**,**b**): PCoA analysis of fecal BAs profile at week 3 (**a**) or 6 (**b**) in the HFHC−PC, LFLC−PC, and LFLC−NC groups. (**c**): The relative concentration of colonic total CDCA, CA, β-MCA, LCA, DCA, ursodeoxycholic (UDCA), hyodeoxycholic acid (HDCA), and GDCA in the HFHC−PC, LFLC−PC, and LFLC−NC groups at week 6, respectively. (**d**): The relative concentration of feces total CDCA, CA, β-MCA, LCA, DCA, UDCA, HDCA, GDCA, and TLCA in the HFHC−PC, LFLC−PC, and LFLC−NC groups at weeks 3 and 6. (**e**–**k**): Relative mRNA expression of CYP7A1 (**e**), CYP8B1 (**f**), CYP7B1 (**g**), CYP27A1 (**h**), FXR (**i**), and BSEP (**j**) in liver, and ASBT (**k**) in the colon. * *p* < 0.05, ** *p* < 0.01.

We examined BA levels in the colonic and fecal samples of mice. Principal coordinate analysis (PCoA) showed that the HFHC-PC group had different fecal BA clusters compared to the LFLC-PC and LFLC-NC groups at weeks 3 and 6 (Figure 4a,b). Analysis of BA levels in the colon showed increased levels of chenodeoxycholic acid (CDCA) and bile acids (CA) in the HFHC-PC group at week 6 compared to those in the LFLC-PC group, with no significant differences in other BAs (Figure 4c). Although the LFLC-PC group showed similar fecal BA profiles to the LFLC-NC group after weeks 3 and 6, we found that the levels of CDCA, β-muricholic acid (β-MCA), and DCA increased in the fecal samples (Figure 4d). However, the HFHC-PC group showed higher levels of primary and secondary BA metabolism. Specifically, CDCA, β-MCA, and DCA levels increased significantly at weeks 3 and 6. Second, glycoursodeoxycholic acid (GDCA) and taurolithocholic acid (TLCA) levels significantly increased only at week 3 and LCA levels only at week 6 (Figure 4d). Cholecystectomy significantly upregulated liver CYP7B1 and BSEP mRNA expression compared to the LFLC-NC group (Figure 4g,j). Feeding an HFHC diet after cholecystectomy significantly upregulated the liver CYP7A1, CYP8B1, FXR, and BSEP (Figure 4e,f,i,j) and ileum ASBT (Figure 4k) mRNA expression. Furthermore, we observed that neither cholecystectomy nor an HFHC diet resulted in significant differences in liver CYP27a1 mRNA expression (Figure 4h). In conclusion, feeding an HFHC diet after cholecystectomy similarly exacerbates the disturbances in the BA metabolism, resulting in elevated levels of primary and secondary BA metabolism.

### 3.4. Alterations in Gut Microbiota Associated with BA Metabolism

**Figure 5:**
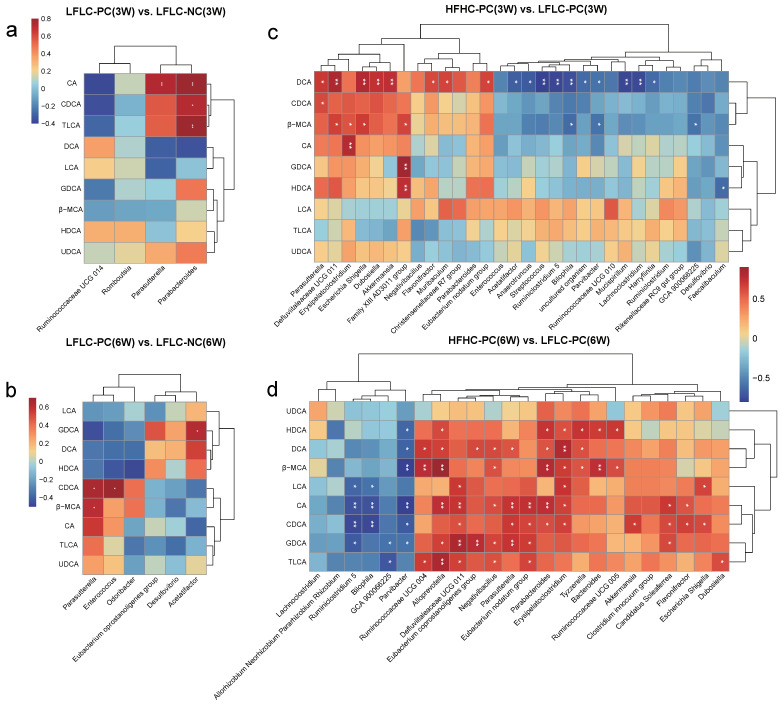
Effect of different diets and PC on BA metabolism in relation to gut microbiota. (**a**,**b**): Spearman’s correlation between gut microbiota and fecal BAs in LFLC−PC vs. LFLC−NC groups at weeks 3 (**a**) and 6 (**b**). (**c**,**d**): Spearman’s correlation between gut microbiota and BAs in HFHC−PC vs. LFLC−PC groups at weeks 3 (**c**) and 6 (**d**). Red denotes a positive correlation; blue denotes a negative correlation. The color intensity is proportional to the strength of the Spearman’s correlation. * *p* ≤ 0.05, ** *p* ≤ 0.01.

Spearman’s correlation analysis was performed to determine the correlation between the differential gut microbiota and fecal BAs. The results showed a significant correlation between gut microbiota and BA metabolism. Specifically, *Parabacteroides* and *Parasutterella* were strongly and positively correlated with altered CA, CDCA, and TLCA in the LFLC-PC group at week 3, respectively (Figure 5a). *Parasutterella*, *Enterococcus*, and *Acetatifacor* were strongly and positively correlated with altered CDCA, β-MCA, and GDCA in the LFLC-PC group at week 6, respectively (Figure 5b). In addition, *Parasutterella*, *Defluviitaleaceae UCG 011*, *Erysipelatoclostridium*, *Escherichia Shigella*, *Family XIII AD3011 group*, *Eubacterium nodatum group*, *Parabacteroides*, *Akkermansia*, *Candidatus Soleaferrea*, *Flavonifractor*, *Alloprevotella*, *Negativibacillus*, *Ruminococcaceae UCG 004*, *Tyzzerella*, *Bacteroides*, and *Ruminococcaceae UCG 005* were strongly positively correlated with alterations in primary BA metabolism (CDCA and β-MCA) in the HFHC-PC group at week 3 or 6 (Figure 5c,d). *Parasutterella*, *Defluviitaleaceae UCG 011*, *Escherichia Shigella*, *Dubosiella*, *Akkermansia*, *Flavonifractor*, *Muribaculum*, *Eubacterium nodatum group*, *Ruminococcaceae UCG 004*, *Alloprevotella*, *Eubacterium coprostanoligenes group*, *Negativibacillus*, *Parabacteroides*, *Erysipelatoclostridium*, *Tyzzerella*, *Escherichia Shigella*, *Candidatus Soleaferrea*, and *Dubosiella* were strongly positively correlated with alterations in secondary BA metabolism (such as LCA, DCA, GDCA, and TLCA) at week 3 or 6 (Figure 5c,d). In summary, we further identified that feeding an HFHC diet after cholecystectomy can disrupt gut microbiota homeostasis and BA metabolism through the gut microbe–BA axis.

### 3.5. HFHC Diet Exacerbates the Effects of Cholecystectomy on the Metabolic Function of the Gut Microbiota

**Figure 6:**
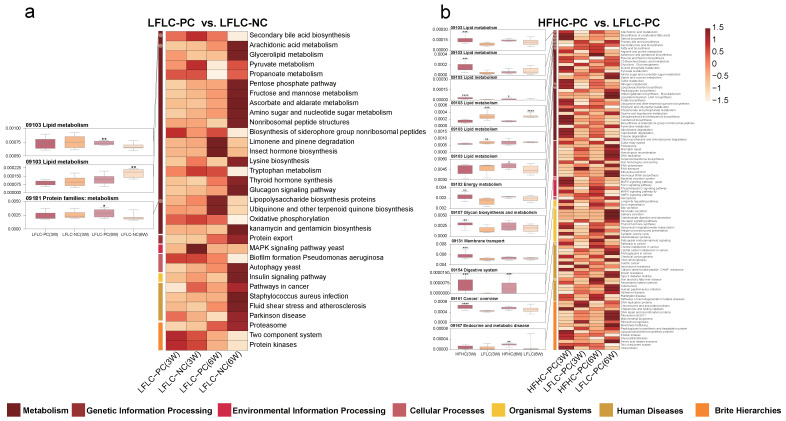
HFHC diets and PC altered gut microbiota metabolism in mice. PICRUSt analysis in the KEGG pathways. (**a**): HFHC−PC vs. LFLC−PC groups (at weeks 3 and 6). (**b**): LFLC−PC vs. LFLC−NC groups (at weeks 3 and 6). The boxplots (**left**) show the relative abundance of the metabolic pathway of the HFHC−PC, LFLC−PC, and LFLC−NC groups (at weeks 3 and 6). All boxplots represent the min to max of the distribution; the median is shown as a thick line in the middle of the box. Heat maps (**right**) show differential metabolic pathways between the two groups (a: top 60 pathways). The color indicates the median relative abundance of the metabolic pathway in the group. Mann–Whitney test was used for comparison between the two groups (*p* < 0.05). * *p* < 0.05, ** *p* < 0.01, *** *p* < 0.001, **** *p* < 0.0001.

The gut microbiota is extensively involved in basic host metabolic activities, and this vast potential function affects whole-body metabolism and is a key factor in altering the metabolic profile of the host. Therefore, we further used PICRUSt predictions to determine the effects of cholecystectomy and feeding the HFHC diet after cholecystectomy on gut microbiota function in mice, and we further analyzed them in the context of the KEGG database (Figures S2a–f and S3a–f). Analysis of differential metabolic pathways showed that feeding an HFHC diet after either cholecystectomy or cholecystectomy significantly affected the metabolic pathways associated with the seven functional classes of the microbiota. Secondary BA biosynthesis and lipopolysaccharide biosynthesis proteins were significantly upregulated, and arachidonic acid metabolism was significantly downregulated in the LFLC-PC group compared to those in the LFLC-NC group (Figure 6a). Feeding an HFHC diet after cholecystectomy resulted in more differential metabolic pathways. Specifically, compared to the LFLC-PC group, the HFHC-PC group showed significantly upregulated arachidonic acid metabolism, biosynthesis of unsaturated fatty acids, steroid biosynthesis, primary BA biosynthesis, fatty acid biosynthesis, nitrogen metabolism, lipopolysaccharide biosynthesis, bacterial secretion system, bile secretion, pathways in cancer, and NAFLD disease metabolic pathways (Figure 6b). In addition, secondary BA biosynthesis was significantly reduced at week 3 and increased at week 6 (Figure 6b). Notably, mismatch repair, DNA repair, and recombination proteins were significantly downregulated (Figure 6b). Functional predictions further revealed that alterations in gut microbiota homeostasis were accompanied by corresponding changes in metabolic function, which may have more profound effects on the host.

### 3.6. HFHC Diet Exacerbates Dysbiosis of Metabolite Metabolism by Cholecystectomy

**Figure 7:**
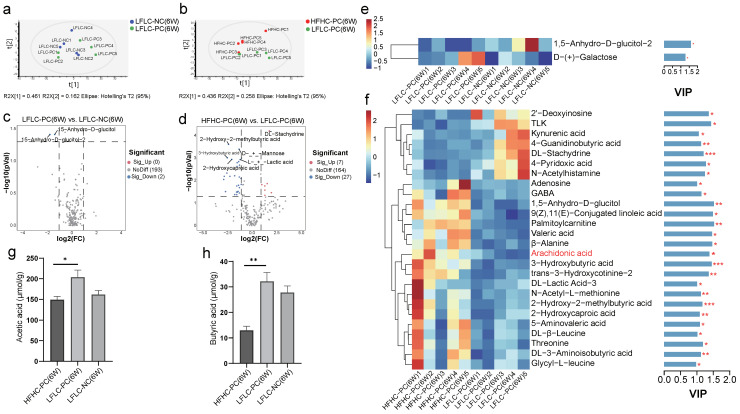
HFHC diet and PC altered the fecal metabolites in mice. (**a**,**b**): Principal component analysis (PCA) at week 6 (LFLC−PC vs. LFLC−NC (**a**); HFHC−PC vs. LFLC−PC (**b**)). (**c**,**d**): Volcanic map of all differential metabolites and known metabolites (*p* < 0.05) (LFLC−PC vs. LFLC−NC (**c**); HFHC−PC vs. LFLC−PC (**d**)). (**e**,**f**): Heat map analysis of fecal differential metabolites at week 6 in HFHC−PC vs. LFLC−PC groups (**e**) and LFLC−PC vs. LFLC−NC (**f**). The color indicates the median relative abundance of the metabolite in the group of samples. The metabolite clustering tree is shown on the left. The metabolite variable importance (VIP) in the projected values indicates the contribution of the metabolite to the difference between the two groups, as shown on the right. Higher VIP values indicate greater differences in the composition of that metabolite between the two groups. The differential metabolites were set as not less than 1. (**g**,**h**): Acetic acid, Butyric acid. The statistical significance (*p*−value) of the differential metabolites is marked on the right of the bar chart. *p* values were determined using a *t*-test. * *p* ≤ 0.05, ** *p* ≤ 0.01, *** *p* ≤ 0.001.

Next, we examined alterations in bacteria-derived metabolites due to microbial changes. At week 6, untargeted metabolomics analysis showed that cholecystectomy had a small effect on bacterial metabolites (Figure 7a,c and Figure S4a,b), whereas feeding an HFHC diet after cholecystectomy significantly altered the metabolite classification levels in mice (Figure 7b,d and Figure S4c,d). Compared to the LFLC-NC group, the differential metabolites in the LFLC-PC group were 1,5-Anhydro-D-glucitol-2 and D-(+)-Galactose at week 6 (Figure 7e). A total of 26 differential metabolites were examined in the HFHC-PC group compared to the LFLC-PC group at week 6, including 19 upregulated and 7 downregulated metabolites (Figure 7f). Importantly, arachidonic acid was significantly enriched in the HFHC-PC group (Figure 7f), and this substance is important for the cytotoxic effects of BAs. Similarly, to clarify the specific relationship between these metabolites and gut microbiota, we used Spearman’s correlation analysis to correlate differential metabolites with differential microbiota. The results showed that alterations in the gut microbiota were accompanied by the upregulation or downregulation of metabolites. Among these, arachidonic acid was strongly and positively correlated with *Parasutterella*, *Negativibacillus*, *Candidatus Soleaferrea*, *Eubacterium nodatum group*, *Defluviitaleaceae UCG 011*, *Erysipelatoclostridium*, *Alloprevotella*, *Parabacteroides*, *Tyzzerella*, *Bacteroides*, and *Flavonifractor*, and it was strongly and negatively correlated with *uncultured organism* and *Parvibacter* (Figure S5). SCFAs are the main product of the breakdown of indigestible carbohydrates by the gut microbiota, and the HFHC-PC group showed a depleted content of acetate acid and butyric acid (Figure 7g,h). The results showed that feeding an HFHC diet after cholecystectomy altered fecal metabolic profiles in mice.

## 4. Discussion

Cholecystectomy is often accompanied by a range of adverse gastrointestinal symptoms (diarrhea and abdominal pain), leading to the development of PC syndrome [35]. Further epidemiological investigations have suggested that cholecystectomy may increase the risk of diseases, such as CRC [5] and NAFLD [6]. However, the risk of cholecystectomy may be associated with the dysbiosis of gut microbiota and disturbances of the BA metabolism following cholecystectomy [7]. Diet is a key factor that influences BA metabolism and microbial homeostasis [36]. Previous studies have shown that an HFHC diet is positively associated with the development of PC syndrome [9,37] and NAFLD [38] and negatively associated with the consumption of whole grains, legumes, fish, and vegetables. In addition, a population-based cohort study of 1,033,955 also showed that the risk of cholecystectomy was associated with a high intake of ham, whereas adherence to a diet rich in fruits, vegetables, legumes, and olive oil was associated with a reduced risk of cholecystectomy [8]. In summary, an HFHC diet after cholecystectomy is a potential factor that increases the risk of cholecystectomy. However, whether high dietary cholesterol intake exacerbates disturbances in BA metabolism and disrupts gut microbial homeostasis after cholecystectomy remains unknown.

Based on this, we successfully constructed a cholecystectomized mice model and fed diets with different levels of fat and cholesterol. The results showed that cholecystectomy significantly increased the expression levels of colonic IL-10, whereas NF-κB showed an increasing trend. However, an HFHC diet after cholecystectomy further increased the levels of the colonic pro-inflammatory cytokines IL-1β, TNF-α, IL-6, and NF-κB, although no significant damage could be detected in histology. Our study suggests that the intake of an HFHC diet after cholecystectomy increases the risk of intestinal pro-inflammation.

Furthermore, we clarified the possible factors of an HFHC diet that promote inflammation after cholecystectomy. The gut microbiota can influence host health through its direct involvement in immune and metabolic pathways [39]. The results showed that an HFHC diet after cholecystectomy exacerbated the disruptive effects of cholecystectomy on the structural and metabolic functions of the gut microbiota. During the experimental period, we found that the effect of cholecystectomy on the structure of the gut microbiota was largely dependent on time, which altered the beta diversity at week 6. In addition, feeding the HFHC diet after cholecystectomy significantly increased the alpha diversity (week 6) and altered the beta diversity (weeks 3 and 6) of the microbiota. Although decreased microbial diversity is associated with several diseases [40], higher diversity does not necessarily imply a healthier microbial community. This may be due to the fact that the HFHC diet promoted the colonization of species that have damaging effects on the intestinal barrier [41]. Interestingly, the ratio of Firmicutes to Bacteroides (F/B), a well-known biomarker of obesity, showed an increasing trend in many HFHC studies [42]. However, in our study, the F/B ratio in the HFHC-PC group showed a decreasing trend at both weeks 3 and 6 compared to that in the LFLC-PC group. Further analysis revealed that some representative genera of the phylum Bacteroides (such as *Bacteroides* and *Parabacteroides*) were significantly enriched in the HFHC-PC group and that they all had the ability to metabolize BAs [43], thus leading to a decreasing trend in the F/B ratio.

We further analyzed how the gut microbiota was altered at the genus level. Simultaneously, the abundances of some pathogenic bacteria with pro-inflammatory effects were significantly higher in the HFHC-PC group, such as *Defluviitaleaceae UCG 011*, *Flavonifractor*, *Erysipelatoclostridium*, and *Tyzzerella*. These results were consistent with those of previous studies on HFHC diets [44,45]. It has been hypothesized that they act either directly through secreted products or through the activation of the immune system. In addition, feeding an HFHC diet after cholecystectomy further increased the ability of cholecystectomy to enrich the microbiota associated with BA metabolism. This also suggests that unlike normal mice fed an HFHC diet, PC is also a key factor in the disruption of gut microbiota homeostasis by an HFHC diet. Studies have shown that *Enterococcus*, *Bacteroides*, and *Parabacteroides* possess BSH activity [43]. BSH activity is the main condition for the involvement of the intestinal microbiota in the BA deconjugation reaction, a process that fractures the C-24 N-acyl amide in conjugated BAs and leads to the formation of unconjugated BAs [46]. Moreover, elevated BSH levels are associated with increased bile toxicity [47]. According to reports, *Parasutterella* [48], *Odoribacter* [49], *Dubosiella* [50], *Family XIII AD3011 group* [51], *Christensenellaceae R7 group* [52], *Eubacterium coprostanoligenes group* [53], and *Alloprevotella* [54] can also regulate BA metabolism through direct colonization or indirect action. In our Spearman’s correlation analysis, we further confirmed that altered levels of BA metabolizing genera were the main cause of elevated fecal secondary BA levels. The abundance of *Akkermansia*, a next-generation probiotic, has been reported to be significantly reduced in high-fat diets [55]. However, in our study, *Akkermansia* was significantly enriched by feeding an HFHC diet after cholecystectomy. Studies have demonstrated that *Akkermansia* can regulate BA metabolism by acting directly or by influencing metabolite levels [56,57]. Therefore, HFHC feeding after cholecystectomy may enrich *Akkermansia* by upregulating the BA metabolism pathway. Notably, excessive intestinal *Akkermansia* counts have been reported to damage the intestinal barrier and cause elevated pro-inflammatory cytokine expression [58]. SCFAs are products of dietary fiber fermentation by the gut microbiota, are involved in host immune regulation, and have anti-inflammatory effects. We identified several major SCFA producers in the HFHC-PC group, including *Lachnoclostridium* [59], *Fecalibaculum* [60], *Anaerotruncus* [61], and *Ruminiclostridium* [62]. These results are consistent with the fecal levels of fecal SCFAs, as feeding an HFHC diet after cholecystectomy significantly reduced the fecal levels of acetate and butyric acid. This is detrimental to the intestine and may also contribute to elevated pro-inflammatory levels in the intestine.

The gut microbiota–BA axis is the main pathway of the BA metabolism, and studies have shown that microbial diversity significantly influences the BA pool size [12]. We found that cholecystectomy only upregulated liver CYP7B1 and BSEP mRNA expression levels, whereas an HFHC diet after cholecystectomy upregulated liver CYP7A1, CYP8B1, FXR, and BSEP and ileal ASBT mRNA expression levels. This leads to further accumulation of primary and secondary BAs in the feces of those consuming an HFHC diet after cholecystectomy [63]. It consists mainly of the primary BAs CDCA, CA, and β-MCA and the secondary BAs LCA, DCA, GDCA, and TLCA. This may be attributed to increased BA synthesis and an increased number of enterohepatic cycles [64]. Meanwhile, our study suggests that the classical synthesis pathway of hepatic BAs was significantly promoted in the HFHC-PC group. In conclusion, an excessive intake of cholesterol, a precursor of BA synthesis [65], can increase BA accumulation by promoting the upregulation of BA synthase following cholecystectomy.

Owing to their strong hydrophobic properties, BAs have toxic effects on cells. The hydrophobicity of the BAs decreased in the following order: LCA > DCA > CDCA > TDCA > TCDCA [66]. For example, in studies on the mechanisms on cholestatic liver injury, it has been suggested that the intrahepatic accumulation of hydrophobic BAs (such as DCA and CDCA) may be a possible cause of liver injury [67].

Previous studies have reported that high levels of secondary BAs can have adverse effects on the host, mainly by promoting inflammation, oxidative DNA damage, and activation of the NF-κB pathway [16]. First, BAs lead to activation of NF-κB, mainly through direct disruption of the plasma membrane, which, in turn, increases the pro-inflammatory response. This is because activated NF-κB transcribes genes encoding pro-inflammatory cytokines (such as IL-6, IL-1β, and TNF-α) in the cell nucleus [16]. In addition, DCA may lead to the increased production of pro-inflammatory cytokines, in part, by activating pro-inflammatory macrophages to polarize towards the M1 phenotype [16]. Studies have established that the chronic intake of high doses of DCA exacerbates intestinal inflammation and accelerates the transition from intestinal adenoma to colonic adenocarcinoma [68]. In conclusion, our study suggests that intake of an HFHC diet after cholecystectomy increases the metabolism of primary and secondary BAs in the intestine. High levels of BA metabolism can have adverse effects on the intestinal tract when exposed to it for long periods of time.

Perturbation of the gut microbiota and BA metabolism was further confirmed by predicting KEGG metabolic pathways. Cholecystectomy significantly upregulated secondary BA biosynthesis and lipopolysaccharide biosynthesis protein metabolic pathways in the microbiota, a phenomenon similar to that observed in the HFHC-PC group. LPS acts as a pro-inflammatory factor in the cell wall of Gram-negative bacteria and can significantly contribute to a sustained inflammatory response in immunocompromised hosts or when barrier integrity is compromised [69]. In addition, metabolic pathways associated with lipid metabolism, such as arachidonic acid metabolism, primary BA biosynthesis, and fatty acid biosynthesis were also significantly upregulated in the HFHC-PC group, consistent with the high levels of fecal BA metabolism. Importantly, the microbiota in the HFHC-PC group exhibited a lack of genetic information processing capacity compared to that in the LFLC-PC group. Examples include DNA replication, mismatch repair, and homologous recombination metabolic pathways. Among these, loss of function in homologous recombination has been shown to be a key factor in DNA repair failure [70]. In addition, DNA replication and mismatch repair play important roles in genomic stability and tumorigenesis [71]. These findings further confirm that an HFHC diet after cholecystectomy can adversely affect the intestines by disrupting gut microbiota homeostasis and affecting gut microbiota-related metabolic functions.

Untargeted metabolomics has also revealed possible reasons for the formation of a pro-inflammatory environment. We noted that arachidonic acid metabolism levels were significantly higher in the HFHC-PC group than that in the LFLC-PC group. Perturbation of the cell membrane by BAs has been reported to activate cytoplasmic phospholipase A2, which uses cyclooxygenase and lipoxygenase activities to release arachidonic acid from the cell membrane, ultimately increasing intracellular reactive oxygen species (ROS) levels [66]. In addition, TLK, kynurenic acid, 4-guanidinobutyric acid, and dl-stachydrine showed lower levels in the HFHC-PC group. TLK has been reported to play a central role in DNA repair and replication and is a potential target for novel cancer therapies [72]. In addition, kynurenic acid, 4-Guanidinobutyric acid, and dl-stachydrine possess anti-inflammatory activities. Kynurenic acid acts as an activator of various receptors (such as the aryl hydrocarbon receptor and G protein-coupled receptor) and is involved in the host immune response, with anti-inflammatory activity [73]. 4-Guanidinobutyric acid [74] and dl-stachydrine [75] can also maintain intestinal function and prevent inflammation through various molecular mechanisms.

In conclusion, our study showed that an HFHC diet after cholecystectomy promotes intestinal inflammation, mainly through the enrichment of microbiota associated with the BA metabolism and pro-inflammatory effects while decreasing the abundance of major SCFA producers. In addition, alterations in gut microbial homeostasis leading to further accumulation of fecal BAs and pro-inflammatory metabolites are key factors in the development of pro-inflammation.

## 5. Conclusions

Comprehensive multi-omics analyses showed that an HFHC diet after cholecystectomy in our study could further promote intestinal inflammation, and the mechanism of action was related to the exacerbated gut microbiota dysbiosis and BA disorders in cholecystectomy. There are several limitations of our study. We failed to observe significant histological changes during our experimental period. In addition, to clarify the effect of different dietary patterns on intestinal health after cholecystectomy, we set up only the cholecystectomy group in the HFHC dietary pattern. Thus, we were unable to confirm whether similar results were seen in an HFHC dietary model beyond the cholecystectomy model. In the future, we propose to investigate the long-term effects of an HFHC diet on histological changes after cholecystectomy and potential interventions to mitigate the pro-inflammatory effects. In addition, further clarification of whether cholecystectomy is a potential factor in HFHC diets impairing intestinal health could help us to better understand the risks after cholecystectomy. Despite these limitations, our study revealed the mechanisms involved in the promotion of intestinal inflammation by an HFHC diet after cholecystectomy. Long-term dietary patterns and probiotic interventions are key factors in the development of stable gut microbiota homeostasis and metabolic profiles. Targeting the gut microbiota by designing targeted food and probiotic interventions to modulate the gut microbiota with key metabolic functions to maintain a stable pool of BAs after cholecystectomy is key to improving intestinal health after cholecystectomy. In conclusion, our findings provide a theoretical basis for reducing PC risk in future clinical applications.

## Data Availability

All data generated during and/or analyzed are available from the corresponding author on reasonable request.

## Supplementary Materials

The following supporting information can be downloaded at: https://www.mdpi.com/article/10.3390/nu15173829/s1, Figure S1: Microbial co-abundance analysis at genus level. (a,b): Microbial co-abundance analysis at genus level in the LFLC−PC group at weeks 3 (a) and 6 (b). (c,d): Microbial co-abundance analysis at genus level in the HFHC−PC group at weeks 3 (c) and 6 (d). Black line connections represent relevance. The color of the nodes is based on phylum and the size is based on edges connected to the nodes (*p* ≤ 0.05, two-sided tests of 1000 permutations). Only lines corresponding to correlations whose magnitude is greater than 0.1 are drawn. Figure S2 KEGG enrichment analysis in LFLC−PC vs. LFLC−NC groups at 3 weeks (a–c) and 6 weeks(d–f). (a,d): Rich Factor represents the ratio of the number of differentially expressed genes in the pathway to the total number of genes in the pathway. The degree of enrichment is represented by the Rich Factor, the number of genes is represented by the bubble size, and the significance level is represented by the bubble color. (b,e): Statistical histogram of KEGG pathway enrichment numbers. The vertical black font represents the KEGG A class, the color font represents the KEGG B class, and the number of genes in class B is shown on the abscissa. (c,f): The name of each KEGG pathway is shown on the left, and the corresponding *Q* value is shown on the right. Figure S3 KEGG enrichment analysis in HFHC−PC vs. LFLC−PC groups at 3 weeks (a–c) and 6 weeks (d–f). (a,d): Rich Factor represents the ratio of the number of differentially expressed genes in the pathway to the total number of genes in the pathway. The degree of enrichment is represented by the Rich Factor, the number of genes is represented by the bubble size, and the significance level is represented by the bubble color. (b,e): Statistical histogram of KEGG pathway enrichment numbers. The vertical black font represents the KEGG A class, the color font represents the KEGG B class, and the number of genes in class B is shown on the abscissa. (c,f): The name of each KEGG pathway is shown on the left, and the corresponding *Q* value is shown on the right. Figure S4 Metabolomics analysis of fecal samples from mice in LFLC−PC vs. LFLC−NC groups at 6 weeks (a-b), and HFHC-PC vs. LFLC-PC groups at 6 weeks (c-d). Partial least squares discriminant analysis (PLS-DA) and orthogonal partial least squares discriminant analysis (OPLS−DA). Figure S5 Spearman analysis of differential metabolite and genus genus correlation (HFHC−PC vs. LFLC−PC at 6 weeks). * *p* ≤ 0.05, ** *p* ≤ 0.01. Table S1. The feed formulation details. Table S2. The elution gradien. Table S3. Quantitative Real−Time Polymerase Chain Reaction (qRT−PCR) primer.
