# Supplementary material for: A High-Fat, High-Cholesterol Diet Promotes Intestinal Inflammation by Exacerbating Gut Microbiome Dysbiosis and Bile Acid Disorders in Cholecystectomy
Source: Nutrients. 2023 Sep 1;15(17):3829. doi: 10.3390/nu15173829 (PMC10489946; doi:10.3390/nu15173829)
Supplement: Supplementary file 1 [file nutrients-15-03829-s001.zip › nutrients-2561639-supplementary.pdf]

Supplementary Materials

1. Methods and materials

1.1 Animal

Table S1. The feed formulation details.

Ingredients	High fat and high cholesterol (g/kg)	Low fat and low cholesterol (g/kg)
Protein	197	141
Carbohydrate	314	707
Fat	335	40
Fiber	70	50
Mineral/Vitamin/choline mixture	66	61
Antioxidant(TBHQ)	0.07	0.01
Cholesterol	18	2
Energy calculated(total):		
Energy density, kcal/g	5	3.6
Energy distribution, %kcal:		
Protein	14%	14%
Carbohydrate	26%	76%
Fat	60%	10%
Total	100%	100%

1.2 Histological analysis

Tissue specimens obtained from the colon were fixed in 4% paraformaldehyde for more than 24h, embedded in paraffin and sectioned at a thickness of 4μm using a slicer. The sections were sequentially placed in environmentally friendly dewaxing solution I (20 min), environmentally friendly dewaxing solution II (20 min), anhydrous ethanol I (5 min), anhydrous ethanol II (5 min), and 75% alcohol (5 min), followed by washing with water. The sections were stained with hematoxylin and eosin (H&E) and examined microscopically.

1.3 Metagenomic sequencing

DNA was extracted from the samples according to the Earth Microbiome Project (EMP) standard protocols using the FastDNA Spin Kit (MP Biomedicals Ltd., Santa Ana, CA, USA). Subsequently, the V3-V4 hypervariable region of the 16S rRNA gene

was PCR-amplified using the universal bacterial primers(341F/806R) and purified using TIANgel Mini Purification Kit (TIANGEN, Beijing, China).

1.4 BAs Measurements

Table S2. The elution gradien.

Time (min)	A	B
0	80%	20%
0-6	80-40%	20-60%
6-25	0%	100%
25-26	0%	100%
26-28	0-50%	100-50%
28-30	50-80%	50-20%
30-32	80%	20%

Flow rate of 0.3 mL/min, column temperature was maintained at 30℃

1.5 RNA extraction and quantitative real-time PCR(RT-qPCR)

Table S3. Quantitative Real-Time Polymerase Chain Reaction (qRT-PCR) primer.

Gene	Forward (5'-3')	Reverse (5'-3')
IL-6	ACCAGAGGAAATTTTCAATAGGC	TGATGCACTTGCAGAAAACA
IL-10	GCTCTTACTGACTGGCATGAG	CGCAGCTCTAGGAGCATGTG
NF-κB	AGCTTCAGAATGGCAGAAGATGA	CAGTGCCATCTGTGGTTGAAATA
CYP7A1	GGGATTGCTGTGGTAGTGAGC	GGTTATGGAATCAACCCGTTGT
CYP8B1	CCTCTGGACAAGGGTTTTGTG	GCACCGTGAAGACATCCCC
CYP7B1	GGAGCCACGACCCTAGATG	GCCATGCCAAGATAAGGAAGC
CYP27A1	GCCTCACCTATGGGATCTTCA	TCAAAGCCTGACGCAGATG
FXR	GCTTGATGTGCTACAAAAGCT	CGTGGTGATGGTTGAATGTCC
BSEP	ATGTGACCTTCCATTATCCTTCTCG	GCTGTAGTGCTGTGCTCTTCC
ASBT	ACCACTTGCTCCACACTGCTT	CGTTCCTGAGTCAACCCACAT
Gapdh	GCAAAGTGGAGATTGTTGCCAT	CCTTGACTGTGCCGTTGAATTT

1.6 Untargeted Metabolomics

1.6.1 Metabolite sample preparation

Briefly, fecal samples (approximately 50mg) were weighed, 200 microliters of water were added for homogenization, and 800 microliters of pre-cooled (-20℃) solution (methanol: acetonitrile = (1:1, v/v)) were added for protein precipitation, and

vortex for 30s. They were then treated with ultrasound in an ice bath (4℃) for 30 minutes. The samples were incubated at -20℃ for 1 hour for secondary precipitation to remove proteins. The obtained supernatant was transferred to a new microfuge tube by centrifugation at a high speed of 15,000rpm for 15 min at 4℃. Samples were concentrated using a SpeedVac system (Thermo Fisher Scientific, MA, USA) and vacuum dried. Before analysis, dried samples were reconstituted in 50% acetonitrile (v/v) and transferred to a glass autosampler vial. For quality control purpose, a QC sample was prepared by pooling 10 uL of every sample.

1.6.2 HPLC-MS analysis parameters

The metabolite samples were analyzed with an UltiMate 3000 HPLC system (Thermo Fisher Scientific, MA, USA) coupled to a high-resolution Q Exactive mass spectrometer (Thermo Fisher Scientific, MA, USA). Chromatographic separation was achieved on Waters ACQUITY UPLC BEH Amide (1.7μm , 2.1×100 mm) column. The flow rate was 0.3mL /min. Mobile phase: Phase A was an aqueous solution containing 25 mM CH3COONH4 (ammonium acetate)+25 mM NH4OH (ammonium hydroxide), and phase B was acetonitrile. The gradient elution procedure is: 0 ~ 1 min, 95 % B; 1 ~ 14 min, 95 %~ 65 % B; At 14 ~ 16 min, 65%~ 40% B; 16 ~ 18 min, 40% B; 18-18.1 min, 40%-95 % B; 18.1 ~ 23 min, 95 % B. The sample size was 2 μL. The sample was kept at 15℃ in the automatic sampler and the column temperature was 40℃.

ESI source parameters were set as follows: spray voltage, 3.5 KV in positive or negative modes,

respectively; sheath gas, 35 arb; aux gas, 15 arb; aux gas heater temperature, 320 °C; capillary

temperature, 320 °C. The Q Exactive was run with polarity switching in full scan ddMS2 mode for all samples and QC samples to acquire MS/MS spectra. The full scan parameters were set as: resolution, 70,000; AGC target, 1e6; maximum injection time, 100 ms; scan range, 70–1050 m/z. The ddMS2 scan parameters were set as: resolution, 17,500; AGC target, 1e5; maximum injection time, 50 ms; scan range, 70–1050 m/z;

top N setting, 10; isolation width, 1.5 m/z; collision energy mode, stepped; collision energy type, normalized; normalized collision energy (NCE) at 20, 40, 60 eV; dynamic exclusion duration was set as 7 s for excluding after 1 time.

1.6.3 Untargeted metabolic data analysis

Metabolome data were analyzed using Compound Discoverer 3.3[1]. Multivariate analysis was performed in SIMCA 14.1 software (Umetrics, Umea, Sweden). Partial least squares discriminant analysis (PLS-DA), unsupervised principal component analysis (PCA), and orthogonal partial least squares discriminant analysis (OPLS-DA) were constructed to identify discriminatory features in relevant comparisons. The number of permutation tests was set to 200. Metabolic features with variable importance in projection (VIP, VIP > 1, and $p < 0.05$) were reserved as potential differential biomarkers.

1.7 Determination of short-chain fatty acid contents

The GC-MS system (Shimadzu Corporation, Kyoto, Japan) used helium as the carrier gas. The gas flow rate was 0.89 mL min−1. The samples were heated from 100 °C to 140 °C at 7.5 °C min−1, followed by heating to 200 °C at 60 °C min−1, and maintained for 3 min; stigma pressure, 62.7 kPa; the ion source temperature and interface temperature were 220 °C and 250 °C, respectively, and the sweep setting range was 2–100 m/z.

2. Results

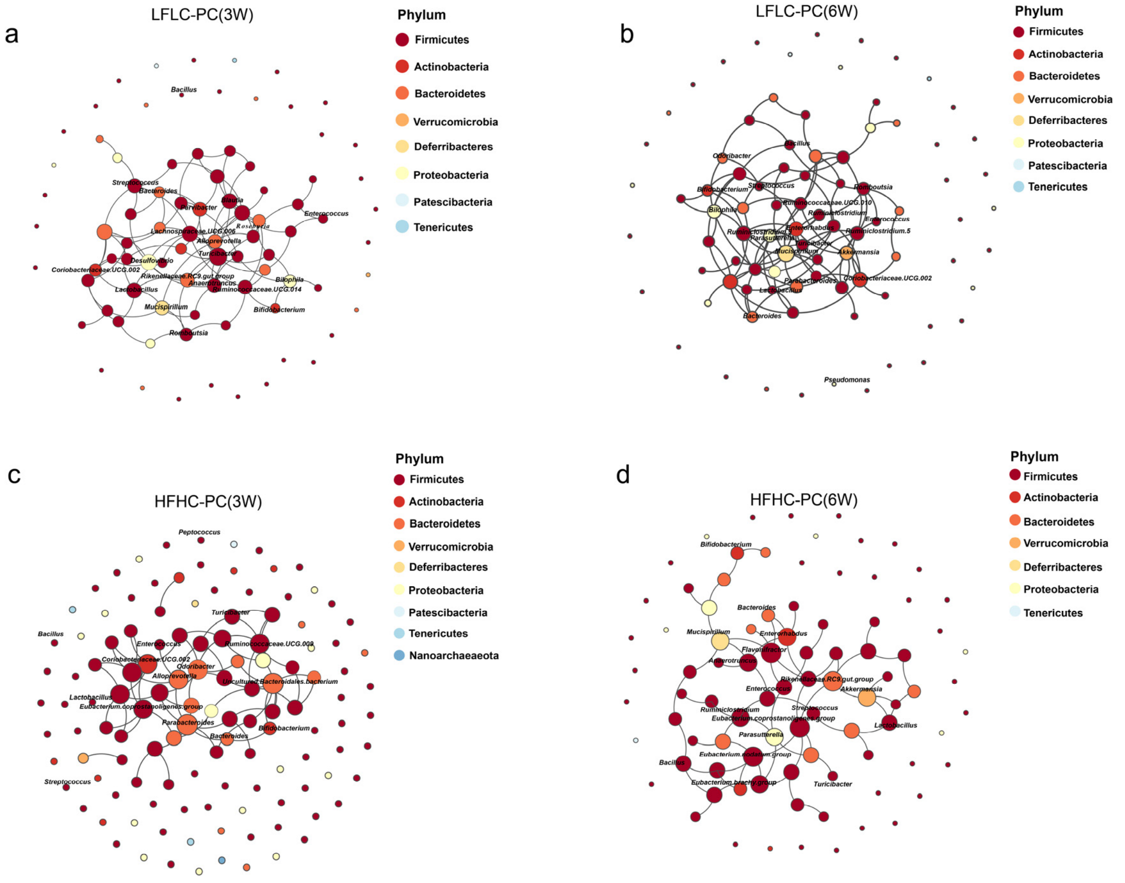

Fig. S1 Microbial co-abundance analysis at genus level. (a,b): Microbial co-abundance analysis at genus level in the LFLC−PC group at weeks 3 (a) and 6 (b). (c,d): Microbial co-abundance analysis at genus level in the HFHC−PC group at weeks 3 (c) and 6 (d). Black line connections represent relevance. The color of the nodes is based on phylum and the size is based on edges connected to the nodes ($p \leq 0.05$, two-sided tests of 1,000 permutations). Only lines corresponding to correlations whose magnitude is greater than 0.1 are drawn.

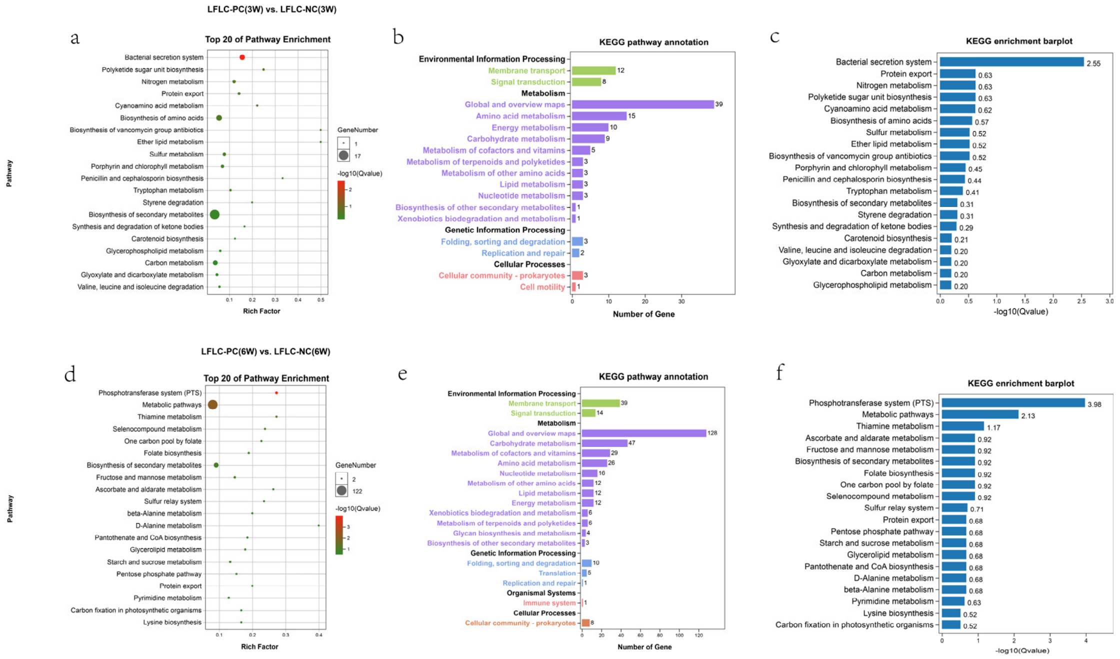

Fig. S2 KEGG enrichment analysis in LFLC−PC vs. LFLC−NC groups at 3 weeks (a−c) and 6 weeks(d-f). (a,d): Rich Factor represents the ratio of the number of differentially expressed genes in the pathway to the total number of genes in the pathway. The degree of enrichment is represented by the Rich Factor, the number of genes is represented by the bubble size, and the significance level is represented by the bubble color. (b,e): Statistical histogram of KEGG pathway enrichment numbers. The vertical black font represents the KEGG A class, the color font represents the KEGG B class, and the number of genes in class B is shown on the abscissa. (c,f): The name of each KEGG pathway is shown on the left, and the corresponding *Q* value is shown on the right.

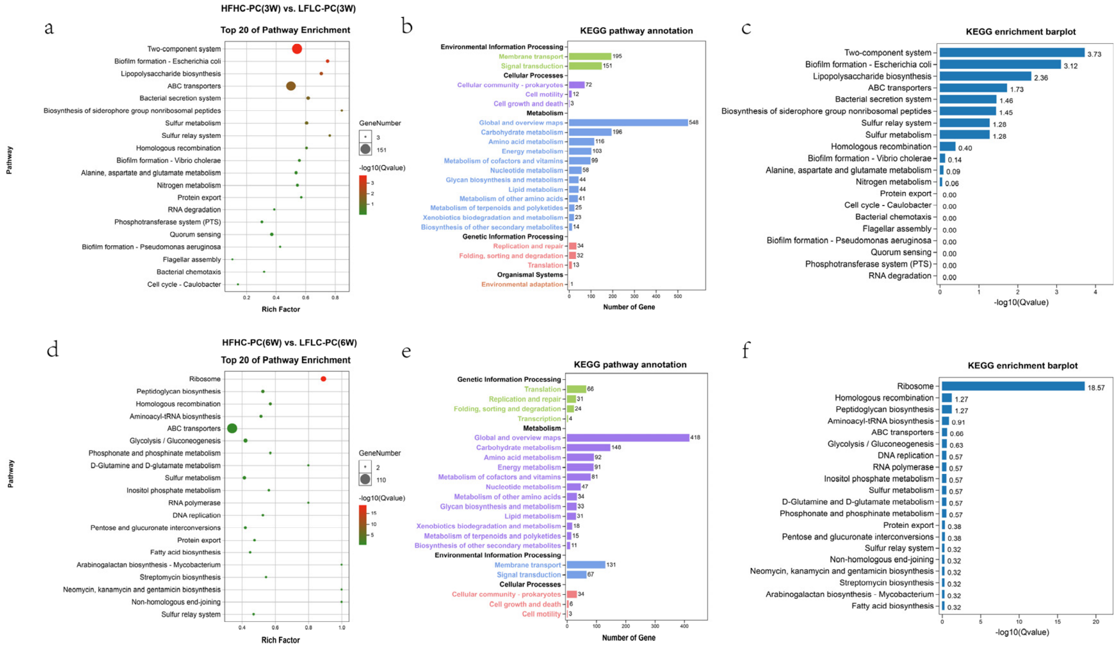

Fig. S3 KEGG enrichment analysis in HFHC−PC vs. LFLC−PC groups at 3 weeks (a-c) and 6 weeks (d-f). (a,d): Rich Factor represents the ratio of the number of differentially expressed genes in the pathway to the total number of genes in the pathway. The degree of enrichment is represented

by the Rich Factor, the number of genes is represented by the bubble size, and the significance level is represented by the bubble color. (b,e): Statistical histogram of KEGG pathway enrichment numbers. The vertical black font represents the KEGG A class, the color font represents the KEGG B class, and the number of genes in class B is shown on the abscissa. (c,f): The name of each KEGG pathway is shown on the left, and the corresponding *Q* value is shown on the right.

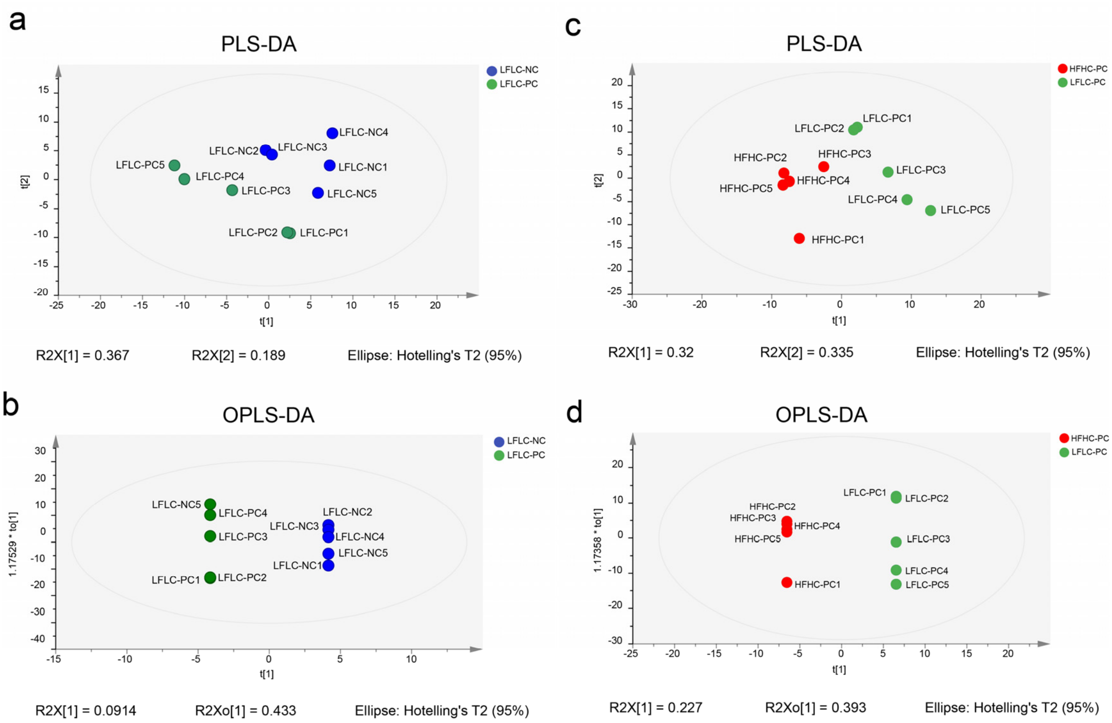

Fig. S4 Metabolomics analysis of fecal samples from mice in LFLC−PC vs. LFLC−NC groups at 6 weeks (a-b), and HFHC-PC vs. LFLC-PC groups at 6 weeks (c-d). Partial least squares discriminant analysis (PLS-DA) and orthogonal partial least squares discriminant analysis (OPLS−DA).

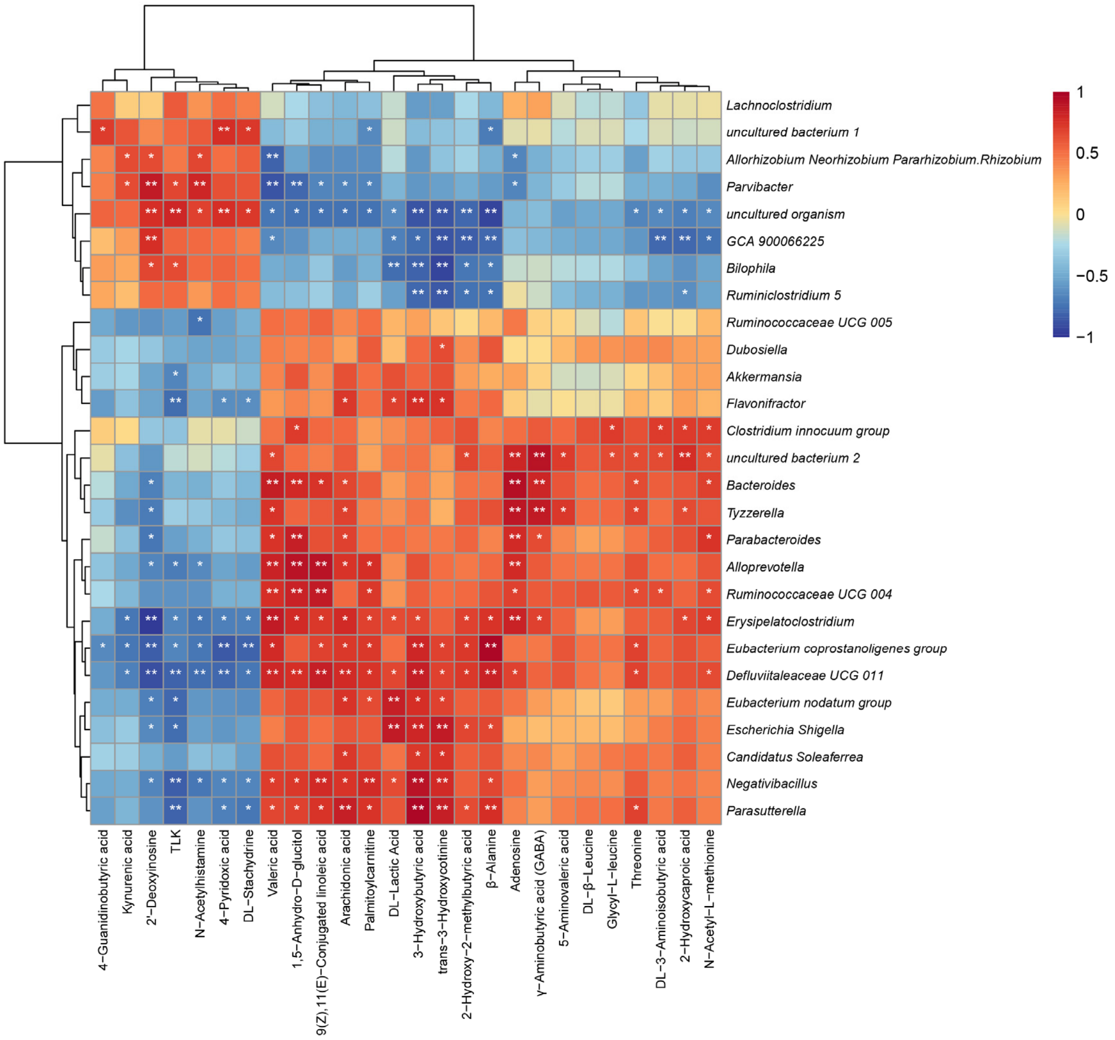

Fig. S5 Spearman analysis of differential metabolite and genus genus correlation (HFHC−PC vs. LFLC−PC at 6 weeks). * $p \leq 0.05$, ** $p \leq 0.01$.

Reference

1. Z. Fan, R. P. Ross, C. Stanton, B. Hou, J. Zhao, H. Zhang, B. Yang and W. Chen, Lactobacillus casei CCFM1074 Alleviates Collagen-Induced Arthritis in Rats via Balancing Treg/Th17 and Modulating the Metabolites and Gut Microbiota, *Front Immunol*, 2021, **12**, 680073.
